# Implementation of surface functionalization of MnS nanoparticles for achieving novel optical properties and improving therapeutic potential

**DOI:** 10.1039/d2ra01087a

**Published:** 2022-07-19

**Authors:** Arpan Bera, Md. Nur Hasan, Nivedita Pan, Ria Ghosh, Reem A. Alsantali, Hatem M. Altass, Rami J. Obaid, Saleh A. Ahmed, Samir Kumar Pal

**Affiliations:** Department of Chemical, Biological & Macromolecular Sciences, S. N. Bose National Centre for Basic Sciences Block JD, Sector-III, Salt Lake Kolkata 700106 India skpal@bose.res.in; Department of Biochemistry University of Calcutta 35, Ballygunge Circular Road Kolkata 700019 India; Department of Pharmaceutical Chemistry, College of Pharmacy, Taif University P.O. Box 11099 Taif 21944 Saudi Arabia; Chemistry Department, Faculty of Applied Science, Umm Al-Qura University Makkah 21955 Saudi Arabia saahmed@uqu.edu.sa; Chemistry Department, Faculty of Science, Assiut University Assiut 71516 Egypt

## Abstract

In the past few years, metal sulfide nanoparticles (NPs) have achieved enormous interest due to their photo and electrochemical properties, which can compete with the existing metal oxide NPs. However, there are fewer reports on the synthesis and the mechanism of surface functionalization of these NPs to achieve intrinsic optical properties. Here, we demonstrate a novel method for the synthesis and the surface modification of manganese sulfide (MnS) NPs to achieve intrinsic photoluminescence and special electrochemical properties. The MnS NPs were characterized using electron microscopy and optical spectroscopic methods. Fourier-transform infrared spectroscopy (FTIR) demonstrated the attachment of citrate on the surface of MnS NPs. The surface modification of insoluble as-prepared MnS NPs by citrate makes them soluble in water. The UV-vis absorption spectra show distinct d–d and ligand to metal charge transfer (LMCT) bands of the citrate-MnS NP nanohybrid. The citrate-MnS NPs exhibited strong photoluminescence. They generated a huge amount of ROS at neutral/acidic pH without any photo-activation which was shown to degrade bilirubin. In addition, the higher ROS generation at pH 5 and pH 7 was exploited to evaluate their anti-bacterial efficacy against *Staphylococcus hominis* (*S. hominis*). These observations could pave the path for the designing and development of new-age surface-functionalized metal sulfide NPs for the benefit of human health.

## Abstract 2

The citrate capping of solubilized MnS nanoparticles in water produced photo-induced pH switching. Citrate-MnS shows remarkable ROS production at acidic and neutral pH in the dark, at pH 5 ROS production demonstrates bilirubin degradation and antimicrobial activity.

## Graphical abstract

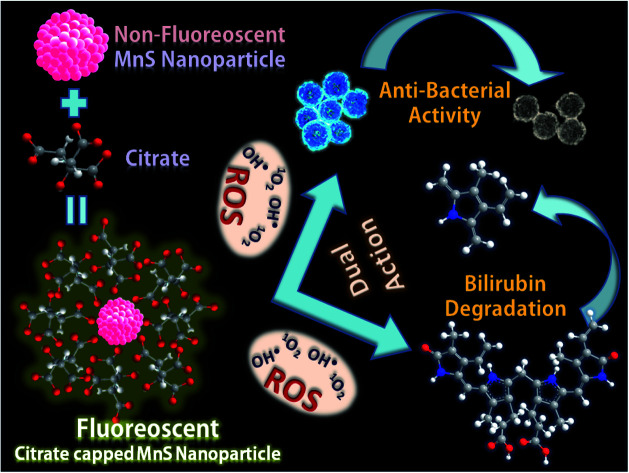

## 1. Introduction

Semiconducting nanoparticles (NPs) have a multitude of unique characteristic properties such as charge transport, light emission, mechanics, and thermal diffusion.^1,2^ Since the last decade, they have represented a dynamic area of interest in molecular and biomedical sciences.^3^ However, NPs tend to form aggregates to minimize their surface energies which creates a technical challenge to control their size, shape, stability, and dispersibility in desired solvents.^4,5^ Therefore, the development of some effective surface protection strategies is very important to maintain the stability of the NPs. These strategies involve functionalization with organic ligands such as small organic molecules or surfactants, polymers, and biomolecules.^6–8^

Over the past decade, 3d transition metal oxide (M_*x*_O_*y*_) NPs have attracted broad interest because of their potential applications in various fields including catalysis, energy storage, drug delivery, and biomedical imaging.^9–13^ In general, most transition metal oxides are analogous to typical transition metal sulfides M_*x*_S_*y*_. Previously, transition metal (Fe, Co, Ni, Cu, Zn, and Mo) sulfides have received much attention due to their manifold crystal structures and potential applications in lithium-ion batteries (LIBs), solar cells, sensors, thermoelectric devices, fuel cells, and supercapacitors.^14–19^ In the past few years, transition metal sulfide NPs have been mainly explored for energy and catalysis-related applications which gained interest from biomedical researchers across the world. However, the use of these NPs for their potential biological applications has still not been elucidated.

Over the last decade, the implementation of surface-functionalized manganese oxide (Mn_*x*_O_*y*_) NPs achieved enormous success in the field of biomedical research, owing to their variable oxidation states (II, III, IV, and VII) which provided a pH-dependent redox medium that has been utilized for its efficient production of ROS and its antioxidant property.^20–23^ In this regard, the development of surface-functionalized MnS NPs could be an exciting attempt to evaluate its potential biological applications. Manganese sulfide (MnS), a p-type semiconductor, has been used for sensing, magnetic resonance imaging (MRI) electrochemical water oxidation, lithium-ion batteries, and photocatalysis because of its excellent optical and electrical properties.^24–29^ However, its biological applications are sparse in the literature. In addition, the consequence of surface functionalization of MnS NPs and the subsequent appearance of novel optical properties have also not been studied yet.

In our present study, we have synthesized MnS NPs and functionalized them with a citrate ligand to formulate citrate-MnS NPs. The surface functionalization made MnS NPs water soluble. Microscopic and optical spectroscopic tools have been used to characterize both uncapped MnS NPs and citrate-MnS NPs respectively. Thermogravimetric analysis (TGA) was done to evaluate thermal stability and loading of citrate on the surface of MnS NPs. We monitored the generation of ROS using dichlorofluorescin (DCFH) indicator. Citrate-MnS NPs produced an excessive amount of ROS at acidic/neutral pH without any photo activation. It is revealed that the mixed valence state of Mn (+2, +3 and +4) in the citrate-MnS NPs led to this exceptional ROS production without photo activation. To study the nature of ROS, we used a singlet oxygen quencher (NaN_3_) and hydroxyl radical quencher (TAB). The citrate-MnS NPs also exhibited a significant efficiency in bilirubin degradation and antibacterial activity against the Gram-positive bacteria *S. hominis*. The cytotoxicity of the as prepared citrate-MnS NPs to evaluate its selectivity to *S. hominis* was assessed through hemolysis assay. This remarkable efficiency of the citrate-MnS NPs in ROS generation without any photo activation indicates its potential therapeutic applications.

## 2. Experimental section

In the present work, all the chemicals were of analytical grade and used without further purification. Manganese acetate (C_4_H_6_MnO_4_), hydrazine (H_4_N_2_), ammonium chloride (NH_4_Cl), thioacetamide (C_2_H_5_NS) and ethylenediaminetetraacetic acid (EDTA) were purchased from Sigma Aldrich. Dimethyl sulfoxide (DMSO) was purchased from Merck. Ultra-pure water was taken from Millipore system (18.2 MΩ cm). Dichlorofluorescein diacetate (DCFH-DA) was obtained from Calbiochem. Sodium azide (NaN_3_) and *t*-butyl alcohol (TBA) were obtained from Sigma-Aldrich.

### 2. 1. Synthesis of bulk MnS NPs

For the synthesis of MnS NPs, manganese acetate (C_4_H_6_MnO_4_) was the source for manganese (Mn^2+^) ions, thioacetamide (C_2_H_5_NS) was used as a source for sulfide (S^−2^) ions, ethylenediaminetetraacetic acid (EDTA) was used as a capping agent, ammonium chloride (NH_4_Cl) was utilized to maintain the pH of the solution and hydrazine (H_4_N_2_) was used as a reducing agent. At first, 5 ml of 2 M ethylenediaminetetraacetic acid (EDTA) solution was added to 10 ml of 1 M manganese acetate (C_4_H_6_MnO_4_) solution in a 100 ml glass beaker and then the mixture was stirred for 5 minutes. After that, 10 ml of 1.4 M NH_4_Cl was added and stirred for another 10 minutes. Then, 0.2 ml of hydrazine (H_4_N_2_) was added to the resulting solution was stirred continuously for 10 minutes. Finally, 10 ml of 1 M thioacetamide (C_2_H_5_NS) solution was added to the solution and stirred for 15 minutes. Immediately, the solution turned pink. The particles were filtered and the filtered particles were washed multiple times with absolute methanol and double distilled water to remove hydrazine. The particles were finally dried at 70 °C for 3 h and stored for further use.

### 2.2. Functionalization of as-prepared MnS NPs by citrate ligand

152 mg of as-prepared MnS NPs was added to 10 ml of 0.5 M sodium citrate solutions in Millipore water under continuous magnetic stirring for 12 hours. Finally, the resulting solution was filtered using a syringe filter of 0.22 μm diameter to filter out the non-functionalized NPs.

### 2.3. Characterization techniques

For the TEM and HRTEM analysis, a diluted solution of citrate-MnS was drop-casted over a carbon-coated copper grid. The particle size was determined from the micrographs recorded using FEI Tecnai TF-20 Feld-emission high resolution transmission electron microscope operating at 200 kV. We do not measure the X-ray diffraction patters. We study the crystal structure and acquire the diffraction pattern through proper procedure. A PAN analytical XPERTPRO diffractometer equipped with Cu Kα radiation (at 40 mA and 40 kV) was used and produced a scanning rate of 0.02° S^−1^ in the 2*θ* range from 20° to 70°. For TGA analysis, uncapped MnS NPs and citrate-MnS NPs were kept under a nitrogen atmosphere. The samples were heated from 30 to 600 °C at a rate of 10 °C min^−1^ by using a PerkinElmer TGA-50H. Absorption and steady-state emission were measured using SHIMADZU spectrophotometer (UV-2600) and a HORIBA Fluorolog respectively. A blue LED source was used (*λ*_max_ = 409 nm and power = 3 mW cm^−2^) was used for light-dependent experiment.

### 2.4. Measurement of ROS

To prepare DCFH, a de-esterification reaction of DCFH-DA was done according to previous literature.^30^ 100 μL of as-prepared citrate-MnS was used for the measurement of ROS. The pH was maintained according to the experiments. Singlet oxygen scavenger NaN_3_ and hydroxyl radical scavenger TBA were used to detect singlet oxygen and hydroxyl radical respectively. 100 μL of as-prepared citrate-MnS was used for the confirmation of hydroxyl radical and singlet oxygen.

### 2.5. Anti-bacterial assay

The antimicrobial activity of citrate-MnS was checked against *S. hominis* bacteria. *S. hominis* were cultured in Luria–Bertani (LB) medium under an incubator shaker of temperature at 37 °C for 24 h. The experiments were done with freshly grown *S. hominis* culture, diluted 10^5^ times and the samples were added on it. The experiments were performed by the colony-forming unit (CFU) assays method. The cells were incubated with 10 μL of as-prepared citrate-MnS NPs at pH 5 and pH 8 separately for 3 h without any photo-activation. Then the cultures were uniformly spread on LB agar plates and the plates were incubated at 37 °C for 24 h to get the CFU.

### 2.6. Bilirubin degradation

For bilirubin degradation, we have used 100 μL of as-prepared citrate-MnS at different pH (pH 5 and pH 7). The solution of bilirubin was prepared at pH 12. After the addition of bilirubin, a clear absorption peak was observed at 432 nm. By monitoring the decrease of absorbance at 432 nm we evaluated the degradation of bilirubin.

### 2.7. Hemolysis assay

We have performed hemolysis assay on RBC cells to evaluate the cytotoxicity of the synthesised citrate-MnS. Mice venous blood (ethical clearance numbers = 02/S/UC-IAEC/01/2019) was collected in a heparinised tube and was divided into three groups, PBS control, negative control and treated. The negative control group comprised of RBC cells incubated in water to ensure 100% hemolysis and in the treated group the citrate-MnS NPs was introduced to the RBC. All the three groups were incubated for 30 minutes and were subjected to centrifugation at 1200 rpm for 10 minutes. The absorbance of the supernatant was taken to obtain the amount of hemolysis.

## 3. Results and discussion

**Fig. 1:**
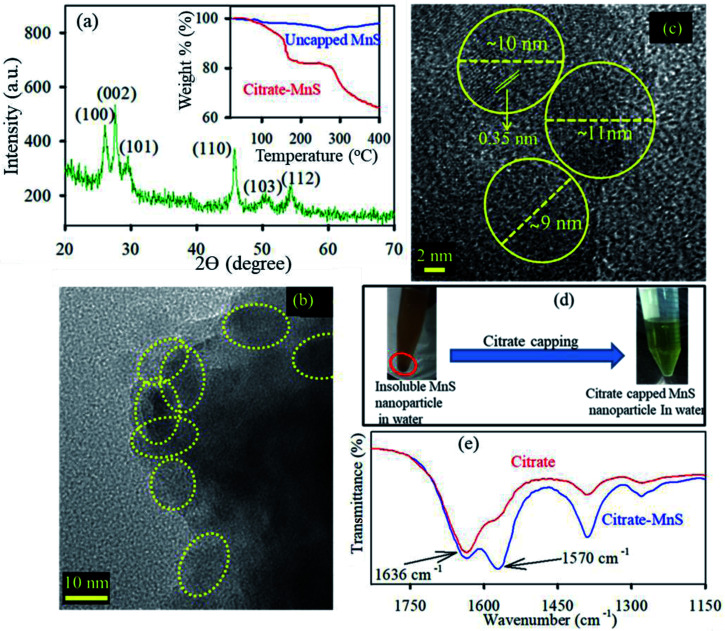
(a) XRD of MnS nanoparticles. Inset shows TGA profile of uncapped MnS NPs (blue) and citrate-MnS NPs (red). (b) and (c) TEM and HRTEM of MnS nanoparticles respectively. (d) Scheme of citrate functionalization with MnS nanoparticles. (e) FTIR spectra of citrate (red) and citrate-MnS (blue).

**Fig. 2:**
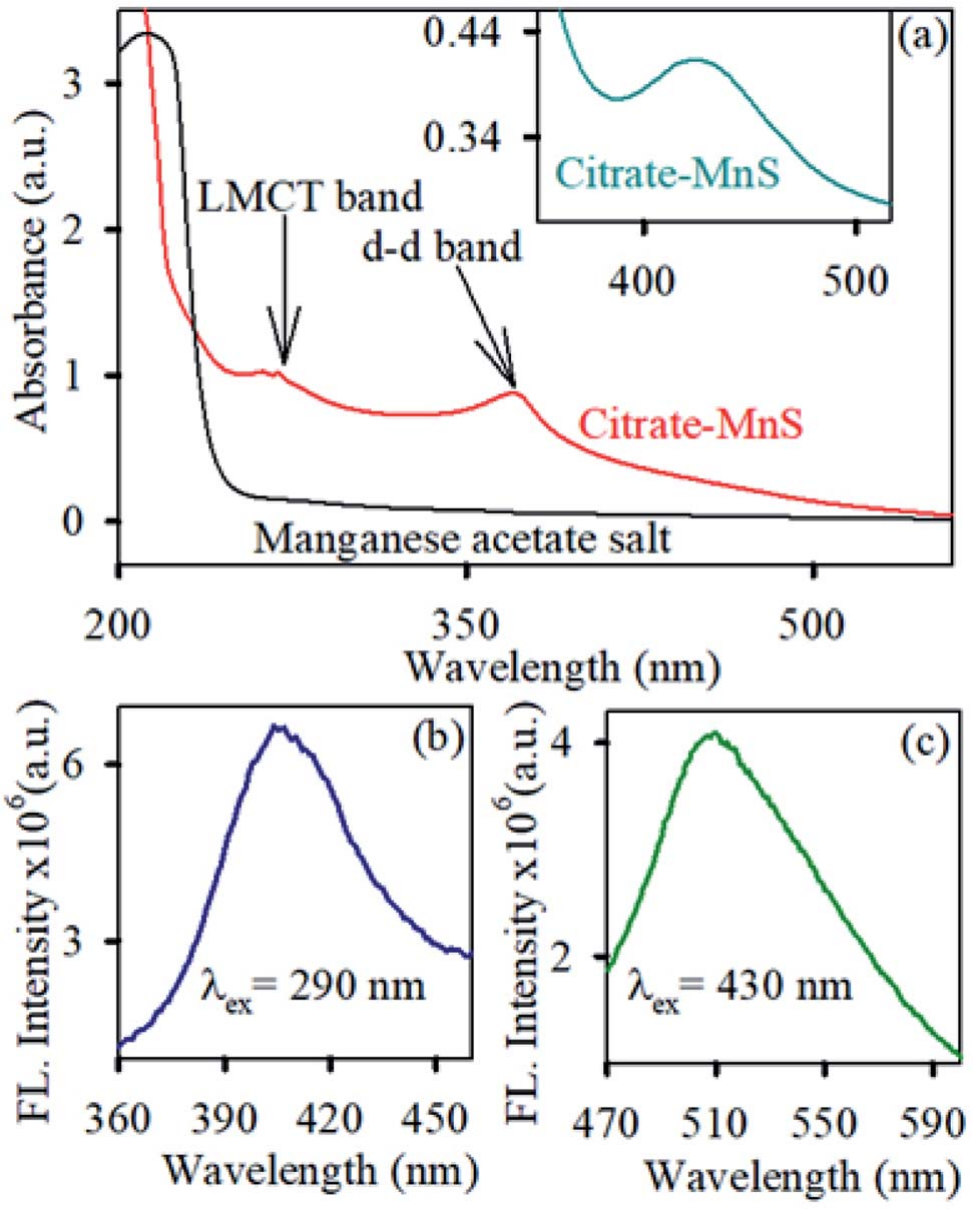
(a) UV-vis absorption spectra of citrate-MnS (red) and manganese acetate salt (black) in water. Inset shows enlarge spectra of citrate-MnS in water (sky blue). (b) and (c) Steady state emission spectra of citrate-MnS upon excitation at 290 nm (blue) and 430 nm (green) respectively.

The diffraction patterns of the as-synthesized NPs are shown in Fig. 1a. All diffraction peaks {(100), (002), (101), (110), (103) and (112) planes corresponding to 26°, 28°, 29°, 46°, 50° and 54° diffraction angles respectively} in the figure perfectly matched with wurtzite MnS crystal indices in the literature.^31,32^ The TEM (Fig. 1b) and HRTEM (Fig. 1c) study reveal the spherical shape and crystalline nature of the MnS NPs. The average size of MnS NPs was found to be around ∼10 nm. The HRTEM image depicts the crystallinity of the MnS NPs having an interplanar distance of 0.35 nm, corresponding to the (100) plane of the crystal lattice.^32^ The functionalization of MnS NPs with citrate influenced their subsequent appearance of novel optical properties. As shown in Fig. 1d, the brownish dispersed solution of MnS NPs turned to a transparent solution of greenish yellow upon citrate functionalization which infers the water solubility of citrate-MnS NPs. The loading of citrate on MnS was studied by thermogravimetric analysis (TGA). The inset of Fig. 1a shows the thermogravimetric curves of uncapped MnS NPs and citrate-MnS NPs. The presence of water molecules is responsible for the initial weight loss up to 154 °C for citrate-MnS NPs. However, a significant percentage of weight loss is observed in between 154 °C to 400 °C for citrate-MnS. The thermal degradation of about 29.7% in between 154 °C to 400 °C for citrate-MnS attributes the presence of citrate molecules. Whereas in case of uncapped MnS NPs, negligible thermal degradation is observed upto 400 °C. The number of citrate molecules on the surface of a single MnS NP is calculated to be 2350. The FTIR spectra of sodium citrate possess stretching frequencies at 1570 cm^−1^ and 1632 cm^−1^ for C–O bonds of CO_2_ group (Fig. 1e). However, a significant inversion in intensity between two peaks was observed for citrate-MnS. This perturbation implies the binding of citrate on the surface of MnS NPs. Citrate-MnS exhibits two characteristic absorption peaks at 290 nm and 430 nm in water (Fig. 2a). The peak at around 290 nm corresponds to the high energy ligand-to-metal charge transfer (LMCT) transition. The LMCT originated due to the interaction between the Mn^3+^ centers and the surface bound citrate ligands in the citrate-MnS NPs.^33^ Another band at 430 nm is attributed to d–d transitions of Mn^2+/3+^ ions in citrate–MnS NPs.^33,34^ Steady state emission spectrum of citrate-MnS shows a characteristic peak at 410 nm upon excitation at the LMCT band (*λ*_ex_ = 290 nm) (Fig. 2b). Whereas, the excitation at the d–d band (*λ*_ex_ = 430 nm) provides a strong emission peak at 520 nm (Fig. 2c).

**Fig. 3:**
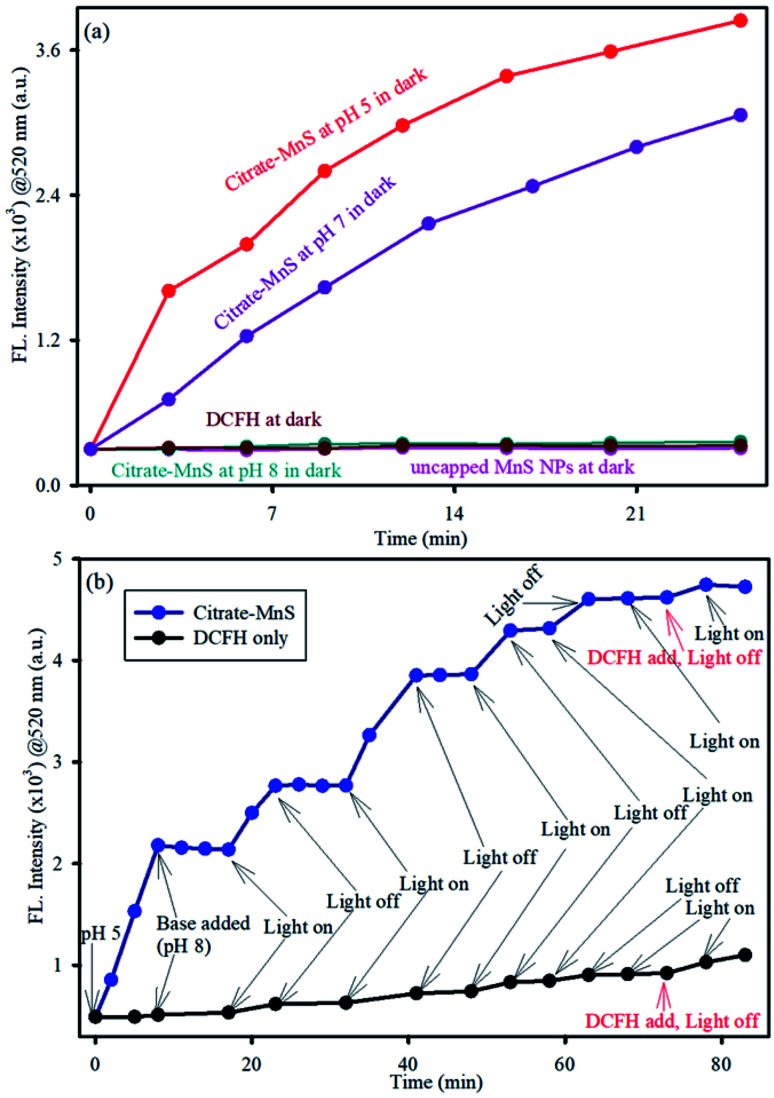
(a) DCFH oxidation (monitored at 520 nm) with time in presence of citrate-MnS at pH 5 (red), at pH 7 (purple), at pH 8 (cyan), uncapped MnS NPs (pink) and DCFH only (gray). (b) DCFH oxidation (monitored at 520 nm) with time in presence of citrate-MnS from pH 5 to pH 8 in a “light on-light off” (blue light) manner with time intervals.

**Fig. 4:**
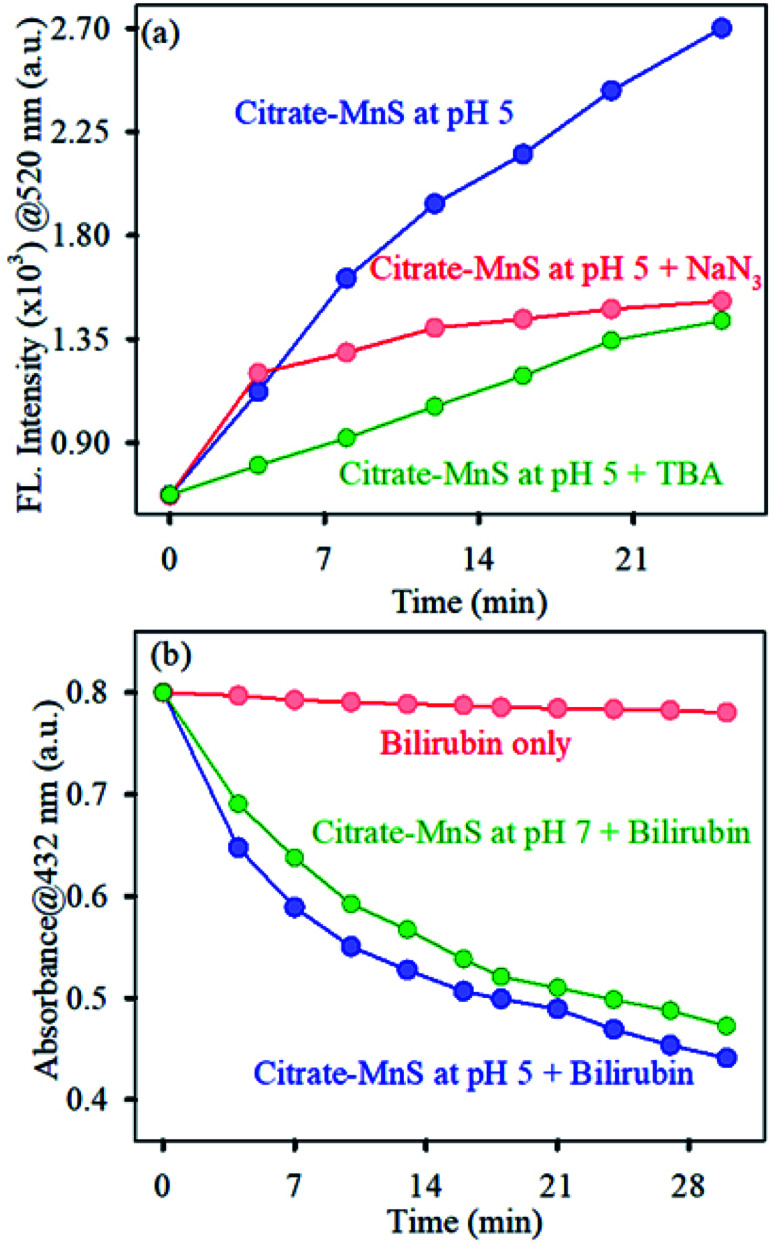
(a) DCFH oxidation (monitored at 520 nm) with time in presence of citrate-MnS at pH 5 with singlet oxygen scavenger NaN_3_ (red), hydroxyl radical scavenger (green) and without any scavenger (red). (b) Bilirubin degradation by citrate-MnS at pH 5 (blue), pH 7 (green) and bilirubin only (red).

To determine the production of ROS by citrate-MnS NPs, a well-known non-fluorescent marker dichlorofluorescein (DCFH) was used which was oxidized to fluorescent dichlorofluorescein (DCF) by ROS. DCF exhibits an emission near 520 nm upon excitation at 488 nm.^35^ The oxidation of DCFH in the dark was monitored for 30 minutes in presence of citrate-MnS at pH 5, pH 7 and pH 8 separately. A huge enhancement of emission intensity at 520 nm at both acidic (pH 5) and neutral (pH 7) pH was observed indicating the presence of ROS and no change in the emission intensity was observed at pH 8 indicating the absence of any ROS generation at pH 8 (Fig. 3a). This phenomenon is consistent with the fact that, in acidic/neutral pH, Mn^3+^ ions are unstable and have the tendency to disproportionate into Mn^2+^ and Mn^4+^.^33^ This redox medium is responsible for a huge generation of ROS.^22,36^ Furthermore, to evaluate the efficacy of pH-dependent ROS generation on photo activation, we performed DCFH assay in the presence and absence of light (Fig. 3b). At first, we measured the ROS at pH 5 under dark for 5 minutes and then a base was added to maintain the pH of the solution at 8. The enhancement of emission intensity at 520 nm was arrested after the addition of the base. But, under blue light irradiation for 5 minutes, the intensity at 520 nm started to increase and further remained constant upon photo deactivation. This process remained continued till saturation was established. The Mn^3+^ ions disproportionate into Mn^2+^ and Mn^4+^ at pH 5 which is responsible for the increment of ROS but the process of disproportionation is interrupted upon addition of base (pH 8). Further, blue light irradiation influences the electron–hole separation in the MnS lattice which in turn, influences the enhancement of ROS production.^37,38^ Furthermore, to investigate the nature of generated ROS, we performed DCFH assay for citrate-MnS at pH 5 in presence of a singlet oxygen quencher (NaN_3_) and hydroxyl radical quencher (TBA).^39,40^ In presence of both NaN_3_ and TBA, the enhancement of emission intensity at 520 nm was significantly quenched which implies the presence of both singlet oxygen and hydroxyl radical as ROS (Fig. 4a). The ability of singlet oxygen production is always very useful in terms of anticancer and antitumor treatment. Moreover, we have performed bilirubin degradation in presence of citrate-MnS at pH 5 and pH 7 for 30 minutes at dark. The decrease in absorbance peak of bilirubin at 432 nm indicates the degradation of bilirubin.^41^ As shown in Fig. 4b, the rate of degradation was significantly higher in both acidic/neutral pH. Whereas, the degradation rate was much higher in the acidic pH as compared to that of neutral pH. The significant production of ROS in acidic/neutral pH is responsible for the degradation of bilirubin. Thus, the citrate-MnS NPs could be implemented as a potential therapeutic agent for hyperbilirubinemia.

**Fig. 5:**
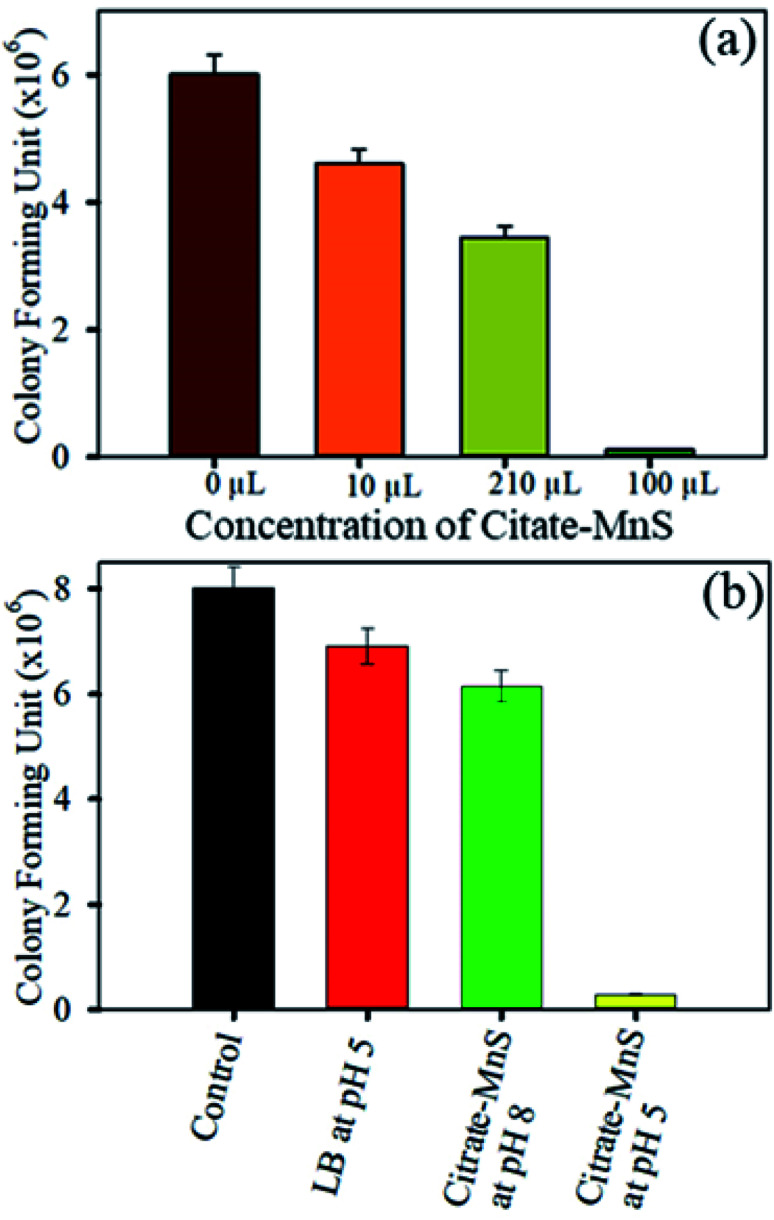
(a) Dose-dependent antibacterial effect of citrate-MnS at concentrations ranging from 0 to 100 μL on *S. hominis* under dark conditions. (b) Bacterial viability after treatment with citrate-MnS at pH 5 and pH 8.

The antimicrobial activity of citrate-MnS was been examined against the *S. hominis* growth to explore the therapeutic potential of citrate-MnS NPs against bacterial infections. A concentration dependent toxicity of citrate-MnS at pH 5 was observed on the growth of *S. hominis* using colony-forming unit (CFU) assay under dark condition. At a concentration of 10 μL, significant inhibition of bacterial growth was observed (Fig. 5a). To probe the antibacterial action 10 μL of as-prepared citrate-MnS was used for incubating the culture of for 3 h. As shown in Fig. 5b, less number of colonies was observed (the bacterial growth is found to be decreased by 96% in CFU) for citrate-MnS treated plate at pH 5 which implies a significant antibacterial effect. Whereas, citrate-MnS treated plate at pH 8 exhibited less inhibition (the bacterial growth is observed to have decreased by 23% in CFU) against the growth of *S. hominis*.

**Fig. 6:**
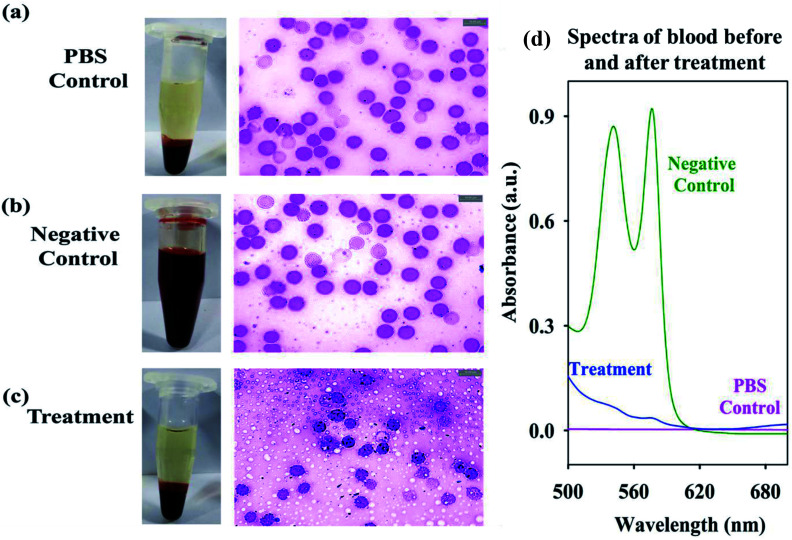
Pictorial diagram for the hemolysis assay for (a) PBS control (b) negative control and (c) NPs treatment. (d) Absorbance spectra of the supernatant before and after NP treatment showing the amount of hemolysis.

To asses the cytotoxicity of the citrate-MnS the hemolysis assay on RBC was performed. We observed no significant hemolysis in the RBC samples treated with the NPs, as comparable to the PBS control group (Fig. 6a and c). On the contrary the 100% hemolysis was observed in the negative control groups due to the rupture of the RBCs 9 (Fig. 6b). To further confirm our results we measured the absorbance of the supernatant (Fig. 6d) which shows absence of any *q* bands of hemoglobin in the treated group. Thus, our experiments suggests that the citrate-MnS NPs are is selective to the microorganisms, having no cytotoxicity to the RBC's. Overall, our study highlights the scope of developing citrate-MnS as a potent antibacterial agent and potential therapeutic agent against hyperbilirubinemia. The ability of pH-triggered ROS production by citrate-MnS could be used for biomedical researchers for utilization of these NPs in potential biological applications.

## 4. Conclusions

In summary, we have for the first time demonstrated that highly water-soluble citrate-MnS NPs exhibit intrinsic photoluminescence and can generate an excessive amount of ROS without any photo-activation. The surface functionalization makes the MnS NPs soluble in water. The citrate-MnS NPs exhibit LMCT and d–d bands and strong photoluminescence properties. FTIR spectra of both capped and uncapped MnS NPs were taken to confirm the surface functionalization of uncapped MnS NPs. DCFH assay was performed to measure ROS without any photo activation. Citrate-MnS NPs decompose bilirubin at acidic pH (pH 5). The pH-dependent disproportionation of manganese ions is responsible for the production of a huge amount of ROS. Detection of singlet oxygen and hydroxyl radical has also been done using an appropriate scavenger. The antibacterial activity of the citrate-MnS NPs is confirmed from the CFU assay on Gram-positive bacteria *S. hominis*. A significant inhibition {(96%) in CFU} was observed for citrate-MnS NPs at pH 5. In addition the NPs showed no cytotoxicity and excellent selectivity to the *S. hominis* bacterial population. The efficacy of ROS production at acidic/neutral pH without any photo activation provides key aspects for multifunctional applications in human health. Therefore, multidisciplinary efforts are needed from biologists to obtain a clear understanding of citrate-MnS-based nanomedicine, which will further facilitate the clinical translation.

## Conflicts of interest

The authors declare no competing financial interest.

## Supplementary Material
